# Epitope determines efficacy of therapeutic anti-Tau antibodies in a functional assay with human Alzheimer Tau

**DOI:** 10.1007/s00401-018-1911-2

**Published:** 2018-09-20

**Authors:** Jean-Philippe Courade, Rachel Angers, Georges Mairet-Coello, Nathalie Pacico, Kerry Tyson, Daniel Lightwood, Rebecca Munro, David McMillan, Robert Griffin, Terry Baker, Dale Starkie, Ruodan Nan, Marta Westwood, Marie-Laetitia Mushikiwabo, Sophie Jung, Geofrey Odede, Berni Sweeney, Andrew Popplewell, Gillian Burgess, Patrick Downey, Martin Citron

**Affiliations:** 1 UCB Biopharma, Chemin du Foriest, 1420 Braine l’Alleud, Belgium; 2 UCB Celltech, 208 Bath Rd, Slough, SL1 3WE UK; 3 Present Address: Vertex Pharmaceuticals, 86-88 Jubilee Avenue, Milton Park, Abingdon, Oxfordshire OX14 4RW UK

**Keywords:** Tau, Tauopathy, Tau antibody, Alzheimer’s disease, Progressive supranuclear palsy

## Abstract

In Alzheimer’s disease (AD) and other tauopathies, the cytosolic protein Tau misfolds and forms intracellular aggregates which accumulate within the brain leading to neurodegeneration. Clinical progression is tightly linked to the progressive spread of Tau pathology throughout the brain, and several lines of evidence suggest that Tau aggregates or “seeds” may propagate pathology by spreading from cell to cell in a “prion like” manner. Accordingly, blocking the spread of extracellular seeds with an antibody could be a viable therapeutic approach. However, as the structure of Tau seeds is unknown, it is only possible to rationally design therapeutic Tau antibodies by making a priori assumptions. To avoid this, we developed a robust and quantitative cell based assay and employed an unbiased screening approach to identify the antibody with the highest activity against human Tau seeds. The selected antibody (D), directed to the mid-region of Tau (amino acids 235–250), potently blocked the seeding of human AD Tau and was also fully efficacious against seeds from progressive supranuclear palsy. When we compared this antibody with previously described reference antibodies, we were surprised to find that none of these antibodies showed comparable efficacy against human pathological seeds. Our data highlight the difficulty of predicting antibody accessible epitopes on pathological Tau seeds and question the potential efficacy of some of the Tau antibodies that are currently in clinical development.

**Electronic supplementary material:**

The online version of this article (10.1007/s00401-018-1911-2) contains supplementary material, which is available to authorized users.

## Introduction

The microtubule-associated protein Tau plays a critical role in the pathogenesis of Alzheimer’s disease (AD) and several related disorders (tauopathies). In these diseases, Tau aggregates and becomes hyperphosphorylated, forming filaments, which can further condense into higher-order neurofibrillary tangles in neurons (for a review see [16]). The development of this pathology is consistently associated with progressive neuronal loss and cognitive decline [39]. Therefore, therapeutics able to block the progression of Tau pathology offer promise as potential treatment options for all tauopathies. In its normal state, Tau is a highly soluble cytoplasmic microtubule-binding protein, which is present in the human central nervous system (CNS) in 6 main isoforms, ranging from 352 to 441 amino acids. Its main role is believed to be the stabilization of microtubules in axons as tracks for axonal transport and as cytoskeletal elements for growth [24]. In AD, neuronal Tau inclusions first appear in the transentorhinal cortex and subsequently spread to the hippocampus and neocortex in a stereotypical manner. A body of recent literature now suggests that the interneuronal transfer of pathological Tau species may drive the spread of pathology in AD [7, 8, 10, 26, 41]. In recent years, several preclinical in vivo tauopathy models have been developed and a number of publications have reported the beneficial effects of active or passive Tau immunization. These studies further support the idea that an extra-cellular Tau intermediate which is accessible to antibodies acts as the key driver in the spread of pathology (for a review see [31]). Based on such data, the first human anti-Tau antibodies have progressed into clinical development.

While there is compelling evidence to suggest that Tau spread underpins the progression of disease, rationally designing a therapeutic to block the spread of Tau is challenging as little is known about the postulated extracellular spreading species. With six splice variants of a 441 amino acid protein that shows a wide variety of posttranslational modifications in disease, in particular hyperphosphorylation, a substantial number of Tau antibodies have been raised against different epitopes. How can one identify the most promising ones?

In this study, we describe how we built a robust and quantitative Tau seed uptake assay in vitro to functionally screen for antibodies that potently neutralize Tau seeds isolated from tauopathy patients. Our data indicate that epitope rather than affinity is the major determinant of efficacy in this paradigm. We suggest that some epitopes may only be present in a fraction of the active seeds, while others may not be present or accessible at all. Therefore, if an antibody is to effectively engage human pathological seeds, targeting the right epitope would appear to be critical.

## Materials and methods

### Peptide generation

All peptides were designed by UCB and synthesized by Peptide Synthetics (Peptide protein Research Ltd., UK).

T159 acetyl-P P G Q K[Ac] G Q A N A T R C*amide

T174 acetyl-C*-K pT P P A P K pT P P amide

T197 acetyl-nY pS pS P cys* pS P G pT P amide

T211 acetyl-R pT P pS L P pT P C * amide

T230 acetyl-R pT P P K pS P pS S C* amide

T254 acetyl- C * K N V K S K I G pS T E amide

T267 acetyl- C * K H Q P G G G K V Q amide

T276 acetyl-Q I I N K[Ac] K[Ac] L D L pS N V Q pS K C* amide

T293 acetyl-pS K D N I K H V P G C * amide

T305 acetyl-C*—pS V Q I V Y K[Ac] P amide

T313 acetyl-V D L S K V T S K C* G pS amide

T335 acetyl-C * V E V K S E K L D amide

T354 acetyl-C*—I G pS L D N I T H V P G G G N K[Ac] K amide

T396 acetyl-C*—pS P V V pS G D pT pS amide

*p* phospho derivative of serine or threonine

K[Ac] = N-epsilon acetyl lysine

*n* nitrosylated

*: point of conjugation for linkage to carrier protein

### Production of recombinant Tau

The gene encoding 2N4R Tau was synthesized by DNA2.0 (ATUM) and cloned into the expression vector pMV-10HisTEV engineered to produce Tau with an N-terminal 10His-TEV tag. Tau protein was expressed using the Expi293TM expression system (Thermo Fisher Scientific) following manufacturer’s instructions. The protein was secreted into the cell culture medium and purified using Ni Sepharose Excel (GE-Healthcare), then cleaved using TEV protease to remove the 10 His tag before further purification using Ni Sepharose Excel. Cleaved 2N4R Tau was collected in the flow through. The protein was then concentrated using an Amicon spin concentrator with a molecular weight cut off of 3000 Da (Millipore) and buffer exchanged into PBS, pH 7.4 to a final concentration of 14 mg/ml.

For recombinant fibril immunization, four different Tau isoforms (2N4R, 1N4R, 0N4R and 0N3R) were expressed in *E. coli* cells. Tau proteins (450 µM) were sterile filtered and shaken in a 1.5-ml Eppendorf tube using a thermomixer (Eppendorf) at 750 rpm, 37 °C for 310 h in PBS, pH 7.4 (0.01 M phosphate buffered saline (NaCl 0.138 M; KCl 0.0027 M); pH 7.4, at 25 °C). Fibril formation was monitored using thioflavin-T dye and reading absorbance on a Fluostar Omega spectrophotometer (BMG Labtech). Fibril formation was confirmed by negative stain electron microscopy.

### Preparation of filamentous Tau-enriched fractions from human brains

Post-mortem human tissues were provided by Asterand Bioscience (now bioIVT, UK). Sample collection and the treatment of all associated data were performed in compliance with appropriate legal and ethical requirements. Asterand utilizes informed consent procedures (from the original donor, next of kin, or legal representative), privacy policies and institutional review board processes. All samples were coded to maintain patient anonymity and Asterand withheld details pertaining to institutions where sample collection occured to maintain full confidentiality. Each sample was accompanied by a clinical report and full neuropathological analysis confirming diagnosis.

Filamentous Tau protein was enriched from AT8-positive brain samples from pools of at least 20 independent donors per preparation with Alzheimer’s disease (AD) or progressive supranuclear palsy (PSP) according to a previously published protocol [24]. Fractions 8 (equivalent to crude PHF-Tau before sucrose gradient centrifugation in this reference) and 11 (equivalent to fraction A2, after sucrose gradient centrifugation in this reference) which have been previously described to be enriched in PHF-Tau were recovered and used for the BIAcore assay (fraction 11) and the cellular assay (fraction 8). Electron microscopy, AT8 immunogold labelling and AT8 western blots were used to confirm PHF recovery in these preparations. Protein concentration was assessed by bicinchoninic acid assay (BCA, Pierce).

### Antibody generation

All animal experiments were conducted according to the UK legislation (Animals Scientific Procedures Act 1986) under a project licence granted by the Home Office.

#### Peptide immunizations

Female New Zealand White rabbits (> 2 kg) were individually immunized sub-cutaneously with the peptides described above (500 µg of peptide was used for each immunization). Prior to initial immunization, all peptides were conjugated to three different maleimide-modified carrier proteins. The first of these, keyhole limpet hemocyanin (KLH), was emulsified in an equal volume of complete Freund’s adjuvant (CFA), for a priming immunization. Rabbits were given 2 booster injections at 21-day intervals using incomplete Freund’s adjuvant (IFA) with ovalbumin (OVA) and then bovine serum albumin (BSA) conjugated peptides, respectively, with bleeds taken from the ear 14 days post immunization.

#### Immunizations with recombinant fibrils and AD-PHF

Female Sprague–Dawley rats (< 180 g) were immunized sub-cutaneously with either 50 µg of recombinant Tau fibrils or 5 μg of fraction 11 AD PHF, emulsified in an equal volume of CFA. Rats were given 3 booster injections at 14-day intervals using IFA, with bleeds taken from the tail vein 14 days post immunization.

For all immunizations, termination occurred 14 days after the final boost and single cell suspensions of spleen, lymph node, bone marrow and peripheral blood mononuclear cells were prepared and frozen in 10% dimethyl sulfoxide/fetal calf serum (DMSO/FCS) at − 80 °C until required for B-cell culturing. B-cell cultures were prepared using the method described previously [43].

Antibody C was derived from an immunization with peptide T197, while antibody D was derived from an immunization with recombinant Tau fibrils

#### Primary screening

The presence of anti-Tau antibodies was detected using a homogeneous fluorescence-based binding assay. Briefly, depending on the immunogen, Superavidin™ beads (Bangs Laboratories) were either coated with the biotinylated Tau peptides (see above) or with soluble or insoluble tau, and anti-Tau-antibody binding was revealed with either a goat anti-rat or a goat anti-rabbit IgG Fcγ-specific Cy-5 conjugate (Jackson). Plates were read on an Applied Biosystems 8200 cellular detection system.

#### Secondary screening

Following primary screening, positive supernatants were consolidated on 96-well bar-coded master plates using an Aviso Onyx hit-picking robot; then B cells in cell culture plates were frozen at − 80 °C. Master plates were then screened in an ELISA assay against Tau peptides described above for peptide antibodies, or 2N4R recombinant Tau for recombinant protein and AD-PHF antibodies. For the ELISA assay, 3 µg/ml peptide or protein was coated onto 384-well Maxisorp plates (Thermo Fisher Scientific/Nunc) in carbonate coating buffer (*d*H_2_O + 0.16% Na_2_CO_3_ + 0.3% NaHCO_3_). Plates were blocked with 1% w/v casein + 1% w/v BSA in PBS and then incubated with 10 μl/well of B-cell culture supernatant. Secondary HRP-conjugated goat anti-rat (recombinant fibrils, AD-PHF) or anti-rabbit (peptides) IgG Fc antibody (Stratech Scientific Ltd/Jackson ImmunoResearch) was added to the plates, followed by visualisation of binding with TMB substrate (3,3′,5,5′-tetramethylbenzidine, from EMD Millipore; 10 μl/well). The optical density was measured at 630 nM using BioTek Synergy 2 microplate reader. B-cell supernatants demonstrating specificity to Tau were selected for variable region recovery.

#### Variable region recovery

To allow recovery of antibody variable region genes from wells of interest, a deconvolution step was performed to enable identification of the antigen-specific B cells in a given well containing a heterogeneous population of B cells. This was achieved using the Fluorescent foci method [5]. Individual B cells, identified using an Olympus microscope, were picked using an Eppendorf micromanipulator and deposited into a PCR tube. Antibody variable region genes were recovered from single cells by reverse transcription (RT)-PCR using heavy and light chain variable region-specific primers. Two rounds of PCR were performed on an Aviso Onyx liquid handling robot, with the nested second PCR incorporating restriction sites at the 3′ and 5′ ends allowing cloning of the variable region into a gamma-1 IgG (VH) or kappa (VL) mammalian expression vector. Heavy- and light chain constructs were co-transfected into HEK-293 cells using Fectin 293 (Invitrogen) and the recombinant antibody was expressed in a 125-ml Erlenmeyer flask in a volume of 30 ml. After 5–7 days of culture, supernatants were harvested and antibodies were purified using affinity chromatography.

The variable region (V-region) sequences for antibodies A, B, E and F were taken from patent applications WO 2014/028777A2, US 2015/0183855 A1, US 2011/0059093 A1 and WO 2016/137811 A1, respectively. Genes encoding the heavy- and light chain V-region sequences for each antibody were designed and constructed using an automated synthesis approach by DNA2.0 Inc. The synthesized genes incorporated restriction endonuclease sites, to facilitate cloning into mouse gamma-1 IgG (VH) or mouse kappa (VL) mammalian expression vectors, with Kozak and N-terminal leader peptide sequences derived from mouse antibody V-region genes, to enable expression in mammalian cells.

The V-region genes for antibody C (from peptide T197 immunization) were cloned into rabbit or murine kappa light chain and IgG heavy chain vectors for mammalian expression, as described above. The V-region genes for antibody D were cloned into mouse kappa light chain and gamma-1 IgG and Fab heavy chain vectors for mammalian expression, as described above.

Recombinant IgGs were expressed in UCB’s proprietary CHOSXE cell line using our electroporation expression platform. Following electroporation (MaxCyte^®^ flow electroporator), cells were cultured in wave bags for 14 days in ProCHO medium (Lonza) containing 2 mM Glutamax, at 32 °C, 5% CO_2_. Culture supernatants were harvested, filtered (0.22 µm) and the IgG antibodies purified by Protein A affinity capture on a MabSelect SuRe TM LX protein A column, followed by gel filtration using a S200 50/60 column (GE Healthcare). Briefly, the filtered lysate was conditioned by the addition of 4 M NaCl and loaded onto the Protein A column, washed with 50 mM glycine glycinate pH 8.8 + 4 M NaCl, followed by 0.15 M sodium phosphate pH 9, and bound antibody was eluted from the column with 0.1 M sodium citrate pH 3.4. The eluate was automatically re-injected onto the gel filtration column with on line neutralization performed during the run and eluted with an isocratic gradient into PBS pH 7.4. Monomer fractions were pooled and filter sterilized (0.22 µm).

### Surface plasmon resonance (SPR): kinetic evaluation of anti-Tau IgGs

Binding assays were carried out at 25 °C using the BIAcore T200 instrument (GE Healthcare). For measurements of extrinsic affinity, AD-PHF fraction 11 was immobilized onto the CM5 chip using amine coupling chemistry. Tau was diluted (1:20 dilution factor) into 10 mM acetic acid (pH 3.5) and multiple manual injections were applied to reach approximately 500 RU of capture. The remaining unbound area was blocked by 1 M ethanolamine pH 8.5.

The HBS-EP + (10 mM HEPES, pH 7.4, 150 mM NaCl, 3 mM EDTA, 0.05% surfactant P20, GE Healthcare) buffer was supplemented with additional 150 mM NaCl and 1.25% CM-Dextran (Sigma) and used as the assay buffer. Two 60-s cycles of 10 mM Glycine (pH 2.0) were used for regeneration. While a flow rate of 10 µl/min was used for immobilization and regeneration, a flow rate of 30 µl/min was used for analyte binding and kinetic measurements.

For kinetic determinations, a concentration range of 1–300 nM of each antibody was prepared (1:3 dilutions) and injected for 180 s and dissociation was monitored for 1200 s. After each sample injection, the surface of Tau was regenerated with two 60-s injections of 10 mM Glycine (pH 2.0). All samples were run in duplicate.

Following background subtraction, binding curves were analyzed using the T200 evaluation software (version 3.0). Kinetic parameters describing intrinsic affinity and avidity of full-length IgG antibodies were determined using the 1:1 binding or bivalent analyte model, respectively.

### Immunohistochemistry

Propath UK Limited provided all human tissue samples and associated data in compliance with appropriate legal and ethical requirements. Propath UK Limited confirmed that the samples were sourced from the collection site with informed consent (from the original donor, next of kin, or legal representative), and that privacy policies and institutional review board processes were followed. All samples were coded to maintain patient anonymity and details pertaining to the collections sites were withheld to preserve donor confidentiality. Formalin-fixed paraffin-embedded (FFPE) human tissues from the cerebral cortex of AD patients were obtained from a commercial provider and 4-µm serial tissue sections made. An indirect immunohistochemistry technique using a semi-automated method on the Ventana Discovery Ultra platform (Roche) was used for all antibodies. FFPE tissue sections underwent deparaffinization and pH 9-based antigen retrieval (Roche). Sections were then incubated for 60 min with the different Tau antibodies diluted to optimized concentrations in Ventana Discovery antibody diluent (Roche). The phosphorylation-specific antibody, AT8, was used to confirm Tau pathology (MN1020, Thermo Fisher Scientific) and negative control isotype mouse IgG1 antibody (MOPC-21, Abcam) was included to confirm the specificity of Tau-antibody staining. OmniMap anti-mouse HRP detection antibody (Roche) was applied for 8 min and Discovery ChromoMap DAB Kit (Roche) was used for automated DAB detection according to the manufacturer’s recommendations. After IHC staining, the slides were washed in EZ Prep solution (Roche) for 1 min. Sections were counterstained with hematoxylin and bluing reagent for 4 min each. Finally, all stained slides were washed, dehydrated and mounted in a permanent mounting medium. Digitally scanned images of stained sections were captured using a NanoZoomer XR system (Hamamatsu) with a 20× objective. All antibodies were tested on brain sections derived from four different AT8-positive AD brains and three different AT8-negative donors.

### Western blotting

Samples were prepared for SDS-PAGE in 1× NuPAGE LDS sample buffer (Thermo Fisher Scientific) and 1× NuPAGE antioxidant (Thermo Fisher Scientific), then heated for 5 min at 95 °C. Proteins were resolved on a 4–12% Bolt SDS-PAGE gel (Thermo Fisher Scientific) in MOPS running buffer (Thermo Fisher Scientific) then transferred to a PVDF membrane (Millipore). The membrane was blocked in Odyssey blocking buffer (Licor), and Tau was detected with Tau5 (Thermo Fisher Scientific), AT8 (Thermo Fisher Scientific) or the antibodies described here, all diluted to 1 μg/ml. Secondary antibodies (Licor) were diluted 1:5000. All washes were performed using PBS plus 0.1% tween 20 (PBS-T). Images were acquired by scanning with a Licor CLX and visualized using Image Studio software.

### Preparation of HEK293F cells expressing human P301S Tau

HEK293F cells (Thermo Fisher Scientific) were transiently transfected with the pcDNA3.1(+) vector expressing human Tau 2N4R with a P301S mutation, using 293Fectin (Thermo Fisher Scientific) according to the manufacturer’s instructions. After overnight growth, aliquots of transfected cells were stored in liquid nitrogen until use.

### Cell seeding

For western blot analysis, HEK293F cells expressing untagged human 2N4R P301S Tau were plated on 6-well dishes at a density of 1,000,000 cells per well in complete 293 expression medium (Thermo Fisher Scientific) containing 10% fetal bovine serum and 1% Penicillin–Streptomycin. Cells were incubated overnight at 37 °C in the presence of 5% CO_2_. The following day, 6 μg (total protein determined by BCA assay) of an AD PHF preparation was combined with 5 μl lipofectamine 2000 in 2 ml of OptiMEM (Thermo Fisher Scientific). Cells were seeded for 3 h, after which mixtures were removed and complete medium was replaced. Cells were incubated 48 h at 37 °C in 5% CO_2_. Lysates were harvested and processed as previously described [11].

### Immunocytochemistry

Soluble Tau was extracted from seeded cells by treatment with 1% Triton X-100 (Sigma) + 4% paraformaldehyde (Thermo Fisher Scientific) in PBS for 20 min at room temperature. Detergent-insoluble Tau was detected with AT8 (Thermo Fisher Scientific, 1:1,000 dilution) and an anti-mouse secondary antibody conjugated to AlexaFluor 488 (Thermo Fisher Scientific, 1:5000 dilution). Confocal images were obtained using a Zeiss LSM880 microscope.

### Antibody testing

To assess antibody efficacy, preparations containing filamentous Tau (50–80 ng/μl total protein) were combined with increasing doses of antibodies in complete cell culture medium and incubated overnight at 4 °C. On the same day, HEK293F cells expressing untagged human Tau (2N4R) with the P301S mutation were plated on poly-d-lysine 96-well plates at a density of 25,000 cells per well. Cells were incubated overnight at 37 °C in the presence of 5% CO_2_. The following day, medium was removed and replaced with Tau-antibody mixtures. Again, cells were incubated overnight at 37 °C in 5% CO_2_. Fresh antibody was added to the cells after 24 h of fibril treatment; then, the cells were allowed to grow for a further 24 h at 37 °C in the presence of 5% CO_2_.

Two days after fibrillar Tau treatment, antibody was removed and cells were washed in PBS. Lysates were prepared using 45 µl of FRET lysis buffer (Cisbio, France) per well and analyzed according to the manufacturer’s recommendations.

### TR-FRET assay

Tau aggregation kits were purchased from Cisbio (France) and used according to manufacturer recommendations, including titration of analyte to ensure measurements were collected in the dynamic range of the assay. Donor and receptor FRET fluorophores were combined according to kit instructions with cell lysate and incubated 24 h at room temperature. Samples were excited at 340 nm and emission values collected at 620 nm and 665 nm using a SpectraMax Paradigm (Molecular Devices). FRET was detected by calculating a ratio of the two emission values (665 nm/620 nm) with Tau aggregation determined as a background-subtracted percent increase over unseeded controls (%Δ*F*). Data were normalized to samples seeded in the absence of antibody and absolute IC_50_ values were calculated from compiled data using the 3-parameter variable slope algorithm of normalized data in Graphpad Prism. One-way ANOVA followed by Dunnett’s post hoc test was used to determine statistical significance in comparison to negative control samples.

## Results

### Immunization and screening

**Fig. 1:**
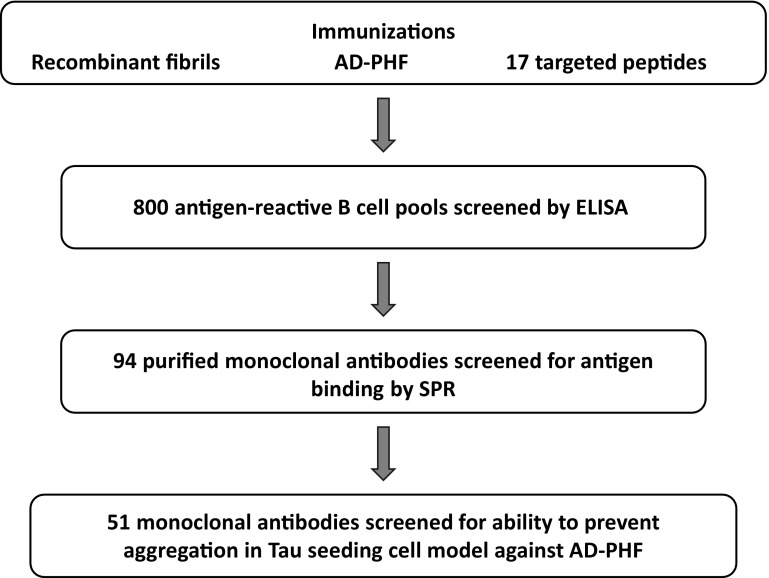
Overview of antibody selection. A diverse set of immunogens was used to generate anti-Tau antibodies. Pools of antigen-reactive B cells were clonally selected initially based on ELISA reactivity, then binding parameters of purified antibodies were characterized by surface plasmon resonance (SPR) using monomeric Tau and AD-PHF Tau. Promising antibodies which exhibited favorable binding kinetics and high affinity were then screened for functional activity in a cell model of seeded aggregation. The most potent antibodies were then selected for further testing

As the epitope requirements of neutralizing Tau antibodies are unknown, we took an unbiased approach in the generation and selection of a therapeutic candidate molecule capable of full and efficient neutralization of seed-competent Tau species. We undertook an extensive immunization campaign utilizing 19 different immunogens including AD-paired helical filaments (PHF) Tau, fibrillar recombinant Tau, and targeted peptides encompassing previously described disease-associated epitopes (Fig. 1). From these efforts, we recovered 800 antigen-reactive B-cell pools. We then performed a preliminary ELISA binding assessment with 217 clones and determined full surface plasmon resonance (SPR) binding kinetics against recombinant and AD-PHF Tau for 94 purified monoclonal anti-Tau antibodies. The most promising candidates were then screened for functional activity against human AD-PHF Tau using a novel seeding cell assay described here. Among the 51 antibodies that were screened for activity, only five displayed robust and complete neutralization of pathological Tau seeds. We selected the most potent and efficacious of these antibodies (antibody D) to take forward and describe it here in direct comparison with other anti-Tau antibodies that have been described in the literature (antibody A [3]; antibody B [42]; antibody E [9]; antibody F [22]). We also generated a high-affinity antibody to Tau phosphorylated at S202 + T205 (antibody C) and describe its functional activity here.

### Characterization of antibody binding parameters

To understand the biological activity of antibodies in the disease environment, it is important to determine their specificity and affinities for monomeric and pathological fibrillar forms. During the course of disease, Tau undergoes a conformational change from an unstructured physiological protein to a fibrillar state accompanied by phosphorylation, nitration, acetylation, ubiquitination, and amino- and carboxy-terminal truncations. Although these modifications are present in mature neurofibrillary tangles, the species of extracellular Tau responsible for interneuronal transit and seeding are unknown. As any combination of these modifications could potentially affect the binding of the antibody to the seeding species, we assessed the binding parameters of all antibodies to both physiological and pathological Tau species.

**Fig. 2:**
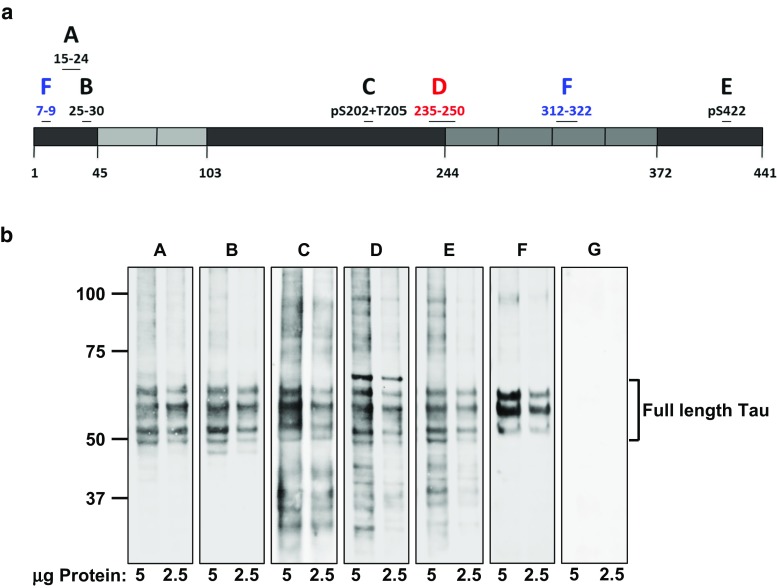
Tau-antibody candidates target different epitopes and differentially recognize full-length and Tau species by Western blot. **a** Antibodies A and B bind epitopes at the N-terminus of Tau, while antibodies C and D bind the proline-rich domain, and antibody E binds a phospho-epitope at the C-terminus. Antibody F recognizes a discontinuous epitope formed when the N-terminus folds into contact the microtubule-binding domain. **b** Sarkosyl-insoluble protein extracts from human AD brains were analyzed by Western blotting with the indicated antibodies. Antibodies directed to the N-terminus of Tau failed to detect small molecular weight cleavage species from sarkosyl-insoluble AD Tau, while antibodies to the central region detected all species. Antibody F detected only full-length Tau species by Western blot, consistent with its discontinuous epitope and N-terminal binding capacity. Antibody G is a control antibody that is not directed against Tau (“Materials and methods”)

Using SPR, we determined the extrinsic affinity of the six full-length immunoglobulin G (IgG) antibodies described here against monomeric Tau and aggregated, hyperphosphorylated PHF Tau isolated from the brains of AD patients (AD-PHF). Despite targeting a variety of epitopes (Fig. 2a), all anti-Tau antibodies bound AD-PHF Tau; however, their extrinsic affinities for AD-PHF Tau varied significantly with *K*_D_ values ranging over four orders of magnitude from 0.12 nM for antibody A to 175.4 nM for antibody E (Table 1). In contrast, only antibodies A, B, D, and F bound monomeric recombinant Tau. This result is consistent with the expected binding profiles of antibodies C and E as both require phosphorylation of residues within their epitopes. Antibodies A and D exhibited *K*_D_ values that were largely unchanged between physiological and pathological Tau suggesting that they recognize epitopes that are unmodified in the disease state. Antibody B exhibited a sevenfold increase in binding to AD-PHF Tau suggesting a preference for disease-associated modifications. Antibody F has been reported to bind to a discontinuous epitope that forms following a conformational change associated with disease [22]. Accordingly, a 4.5-fold improvement in binding was observed with AD-PHF Tau compared to monomeric Tau.

**Table 1:** Characterization of anti-Tau antibodies by surface plasmon resonance

Antibody	Selectivity	Tau monomer			AD-PHF		
		*k*_a_ (1/Ms) × 10^5^	*k*_d_ (1/s) × 10^−5^	*K*_D_ (nM)	*k*_a_ (1/Ms) × 10^5^	*k*_d_ (1/s) × 10^−5^	*K*_D_ (nM)
A	Total tau	3.6	5.2	0.16	2.3	2.5	0.12
B	PHF > monomer	0.5	95.3	20.7	0.4	11.7	3.1
C	pS202 + pT205	No binding	No binding	No binding	0.3	36.7	13.4
D	Total tau	2.1	25.3	1.2	1.4	10.3	0.8
E	pS422	No binding	No binding	No binding	0.4	618.1	175.4
F	PHF > monomer	1.8	2050	116.6	7.3	196	25.8

Binding parameters were determined for anti-Tau antibodies against monomeric Tau and PHF Tau isolated from Alzheimer’s disease brain pools. Antibodies A and D display similar binding profiles against monomeric and PHF Tau, while antibodies B, C, E, and F target Tau species found preferentially in disease

### Assessment of antibody reactivity to human pathological Tau

To confirm the specificity of the Tau antibodies, all were analyzed by Western blotting against PHF Tau extracted from the brains of AD patients (Fig. 2b). In each case, three predominant bands were detected, which correspond to the characteristic triplet banding pattern of PHF Tau isolated from AD brains. These bands are composed of all six alternative splicing isoforms [17] which contain extensive post-translational modifications, including hyperphosphorylation, acquired during the course of disease. In addition to the three main bands, antibodies C, D and E also detected lower molecular weight Tau species that were not labeled by antibodies A, B or F, suggesting that these bands are likely to be N-terminally truncated species. As antibodies A and B bind close to the amino terminus of Tau, they are unable to bind Tau species which may be truncated downstream of amino acids 24 and 30, respectively. Antibody F did not detect N-terminally truncated Tau species. This is in line with the previously described epitope specificity of its well-characterized parent antibody MC-1, which shows preferential binding to conformationally altered Tau present in disease [22]. MC-1 binds amino acids 7–9 in the N-terminus of monomeric Tau; however, its binding affinity is increased upon conformational change in which the N-terminus makes contact with the third microtubule-binding repeat. As expected, the negative isotype control antibody G did not exhibit cross-reactivity with Tau in these experiments.

In addition to full-length and N-terminally truncated Tau species, antibody D also recognized a band slightly larger than the highest expected 68 kDa band of the AD triplet. To confirm binding specificity, a peptide competition experiment for antibody D was performed (Suppl. Figure 1, Online Resource 1). Following incubation with a 100-fold molar excess of a peptide within the epitope of antibody D, all binding of sarkosyl-insoluble AD tau was eliminated.

**Fig. 3:**
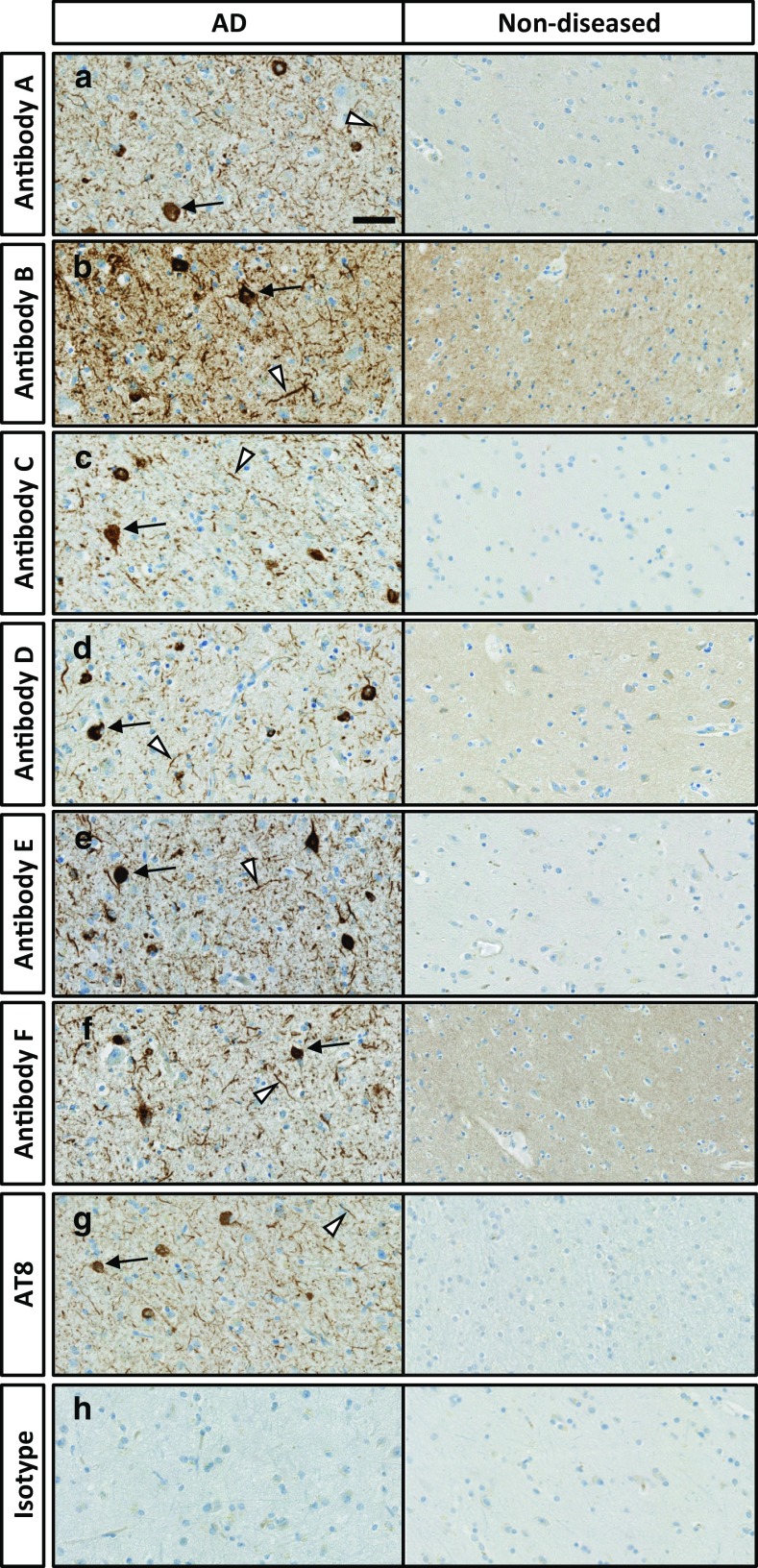
Antibodies directed to different Tau epitopes detect pathological Tau structures in AD brains by immunohistochemistry. **a**–**f** Cortical sections from AD and healthy control (non-diseased) brains were incubated with Tau antibodies A–F, as well as the antibody AT8 (**g**) and a negative control isotype antibody (**h**). All Tau antibodies revealed pathological structures in AD brain sections that were consistent with neurofibrillary tangles (black arrows) and neuritic threads (arrowheads). AT8 immunolabeling is routinely used to identify pathological Tau phosphorylated at Ser 202 and Thr 205 and was employed here to confirm the presence of pathological Tau in the AD brain sample. Tau pathology was absent in the healthy control brain. No apparent labeling was observed with the negative control isotype antibody (**h**) in AD or non-diseased brain tissue. Scale bar 50 μm

The binding of Tau antibodies A–F was also assessed by immunohistochemistry using cortical sections obtained from both AD patients (where Tau pathology was confirmed by AT8 positive immunostaining) and healthy control brains (where no AT8 immunostaining could be detected). All anti-Tau antibodies demonstrated a moderate–strong staining intensity of neuronal bodies and processes in Alzheimer brain tissue (Fig. 3a–f). Numerous positive neuron bodies, dendrites and axons, consistent with neurofibrillary tangles (NFTs) and neuropil threads (NTs), were observed in the AD brain samples that showed positive AT8 immunostaining (Fig. 3g), but were not observed in normal control tissues (Fig. 3a–f) where AT8 signal was absent (Fig. 3g). Neuritic plaques were also observed in sections from AD brains stained with all anti-Tau antibodies. Overall, the strongest intensity and density of positive neuron bodies, dendrites, and axons were observed in samples stained with antibodies B–F. Both staining intensity and density of positive cells/structures were lower in sections stained with antibody A. Finally, no substantial staining was present in AD or healthy control brain sections stained with a negative control isotype antibody (Fig. 3h).

### Development of a seeded aggregation cell model

Multiple independent laboratories have now confirmed the originally unexpected observation that exogenously applied fibrillar proteins can enter naïve cells and induce the misfolding of endogenously expressed homotypic proteins through templated conversion. This phenomenon was first described for prions [6] and has now been demonstrated with multiple proteins including Tau, alpha-synuclein, and SOD1 [18, 28, 33]. Templated misfolding is specific to homotypic proteins and requires a seed composed of the assembled protein of interest and endogenous expression of its monomeric counterpart. Seeded aggregation in cell culture and in vivo is possible using assembled recombinant protein or disease-associated, fibrillar proteins enriched from transgenic mouse or human brains [29, 35].

To test whether Tau antibodies could prevent the development of pathology in recipient cells, we developed a robust and quantifiable cell model of seeded aggregation. To avoid confounding effects resulting from the use of large fluorescent proteins [2], we developed our cell assay using HEK293 cells transiently expressing an untagged full-length (2N4R) Tau construct with the P301S mutation. The cells were bulk transfected in suspension with an efficiency of approximately 85–90% (data not shown). As our aim was to select an antibody capable of neutralizing human pathological tau, we used a standard sarkosyl extraction method to enrich fibrillar Tau from pools of > 20 AD brains for use as seeding material in subsequent experiments.

**Fig. 4:**
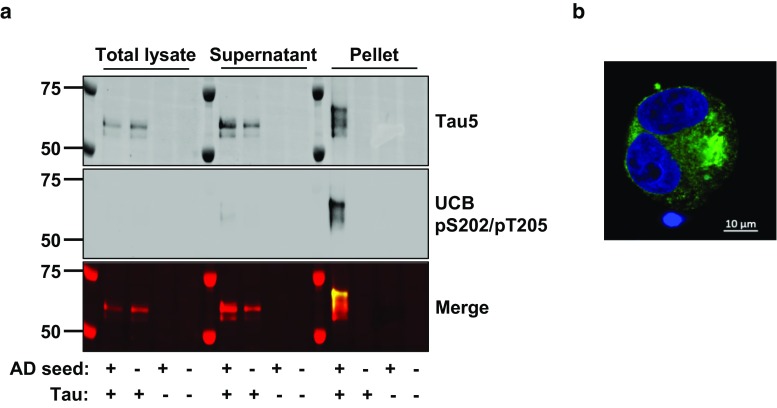
Initial characterization of the seeded aggregation cell model. **a** Only Tau-expressing cells seeded with AD PHF Tau were capable of generating sarkosyl-insoluble, AT8 immunoreactive Tau. The predominant aggregated Tau species present after detergent extraction was hyperphosphorylated and this resulted in a notable increase in molecular weight. Hyperphosphorylation of the insoluble material is demonstrated using UCB’s high-affinity pTau-specific antibody (middle panel). A Tau signal was not observed in unseeded Tau-expressing cells, or vector-only cells treated with AD-PHF Tau. Supernatant and pellet fractions are products from 100,000*g* centrifugation in the presence of 1% sarkosyl. **b** Cells seeded with AD PHF Tau generated small aggregates that appeared to be granular in nature and were labeled with the phosphorylation-specific antibody AT8. Blue labelling: DAPI and green labelling: AT8

Initially, adherent HEK293 cells transfected with P301S Tau or empty vector were treated for 48 h with AD PHF in the presence of Lipofectamine to induce seeded aggregation. Cell lysates were then analyzed by western blot following sarkosyl extraction to establish whether propagated Tau species recapitulated hallmark biochemical features found in human disease. As expected, monomeric Tau from unseeded cells was detected in lysate and high-speed supernatant fractions, but not the detergent-insoluble pellet (Fig. 4a). In contrast, in addition to detecting Tau in lysate and supernatant fractions, Tau-expressing cells seeded with AD PHF generated assembled, hyperphosphorylated Tau which was detected in the sarkosyl-insoluble pellet. Importantly, residual seed material was not observed in vector-only cells, demonstrating that all detectable sarkosyl-insoluble Tau was newly generated during the course of the experiment. Seeded aggregation was also confirmed since only one main hyperphosphorylated species was detected in the sarkosyl-insoluble fraction, rather than three distinct bands present in AD. Hyperphosphorylated Tau was observed by confocal microscopy only after treatment with AD seeds and appeared granular in nature (Fig. 4b), similar to previously reported observations of seeded aggregation using recombinant and transgenic mouse seeds [11, 18].

**Fig. 5:**
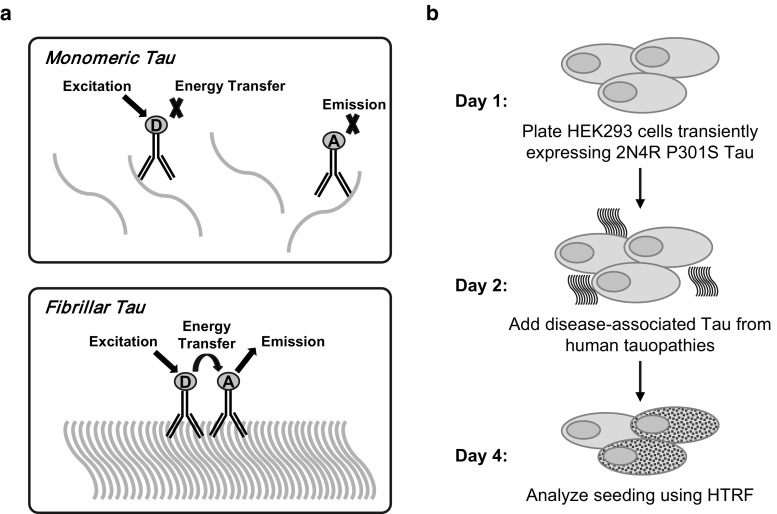
Development of a quantitative Tau seeding cell model. **a** The FRET kit utilizes the same antibody conjugated to donor and acceptor molecules. A positive signal is only generated in the presence of assembled Tau species as the monomeric form contains one epitope, whereas aggregated Tau is polyvalent. **b** HEK293 cells transiently expressing untagged P301S Tau were plated, then treated the following day with disease-associated Tau in the presence or absence of therapeutic candidate anti-Tau antibodies

Since most reports of seeded aggregation with human pathological Tau utilize lipofectamine or other lysosomal disruption agents, we wanted to determine the quantitative limits of this approach and how it might compare with an assay that omitted lipofectamine. To compare methods, we required a robust, quantitative assay to measure assembled Tau species and, therefore, employed a commercially available TR-FRET kit (Cisbio, France). The FRET assay specifically detects assembled Tau species using donor and receptor molecules conjugated to the same antibody (Fig. 5a). As only one epitope is present per Tau monomer, the donor and receptor molecules do not reach a proximity sufficiently close to generate a signal. Assembled Tau, on the other hand, is polyvalent thereby allowing multiple binding events to occur on the same molecule, some of which are close enough to allow energy transfer and signal generation.

Titrations of AD PHF in the presence or absence of lipofectamine (Suppl. Figure 2, Online Resource 1) exhibited marked differences in efficiency. In the absence of lipofectamine, we detected seeding activity when 20–100 ng/μl of total protein was added to cells; whereas in the presence of lipofectamine, seeding was detected from as little as 6 pg/μl through at least 6 ng/μl. When directly compared in the same experiment, we found that the inclusion of lipofectamine increased the seeding efficiency by approximately 2500-fold. As our aim was to assess antibody neutralization activity in solution, we opted to perform all additional experiments in the absence of lipofectamine, despite the fact that this significantly decreased seeding efficiency (Fig. 5b).

**Fig. 6:**
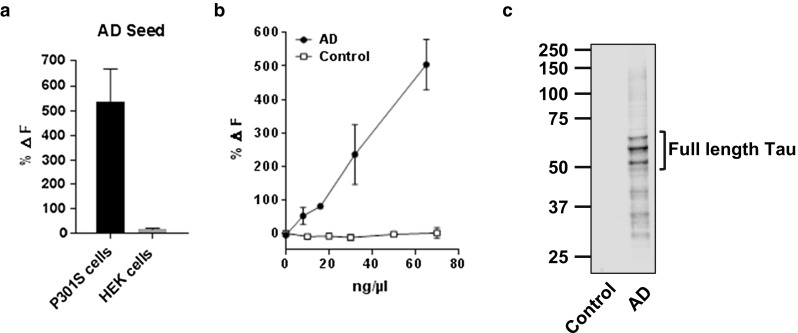
Induction of aggregation requires expression of Tau in recipient cells and a pathological Tau seed. **a** HEK293 cells transfected with P301S Tau or empty vector were treated with extracts from Alzheimer’s disease. In each case, aggregation was detected only in Tau-expressing cells indicating the absence of residual seeding material. **b** To confirm the requirement for aggregated Tau in seeding, a control human brain sample lacking Tau pathology was tested and found to lack seeding activity **c** which is consistent with the absence of assembled, hyperphosphorylated Tau (assessed with AT8). Aggregation efficiency is presented as a background-subtracted percent increase over unseeded cells (%Δ*F*)

Given the large increase in seed concentration required to seed cells without lipofectamine, we performed further validation of the FRET assay using conditions optimized for antibody testing. Cells transfected with empty vector were used as negative controls and confirmed that the FRET signal detected at the end of the assay represented newly induced aggregation and not residual seed material. When treated with AD-PHF seeds, only Tau-expressing cells contained aggregated Tau after 48 h of treatment (Fig. 6a) indicating specific aggregation of overexpressed Tau, rather than the accumulation of exogenous seed material. We also confirmed that the presence of disease-associated Tau in seed material was critical for seeding activity by testing a human brain sample that lacked Tau pathology. In this instance, pathology was not induced as fibrillar Tau seeds were not present in the sample (Fig. 6b, c).

### Assessment of antibody activity against pathological Tau seeds

To select the most efficacious candidate and compare reference molecules, Tau antibodies targeting a variety of physiological and disease-selective epitopes were tested for their ability to neutralize human AD-PHF seeds in a co-incubation paradigm. Dose titrations were performed from 0.1 to 300 nM antibody against optimized concentrations of each seed and added to HEK293 P301S Tau cells. All experiments were repeated at least three times. Using sarkosyl-extracted Tau from seeded cells, we confirmed that the antibodies tested here did not elicit a dose response indicative of interference in the FRET assay (Suppl. Figure 3, Online Resource 1).

**Fig. 7:**
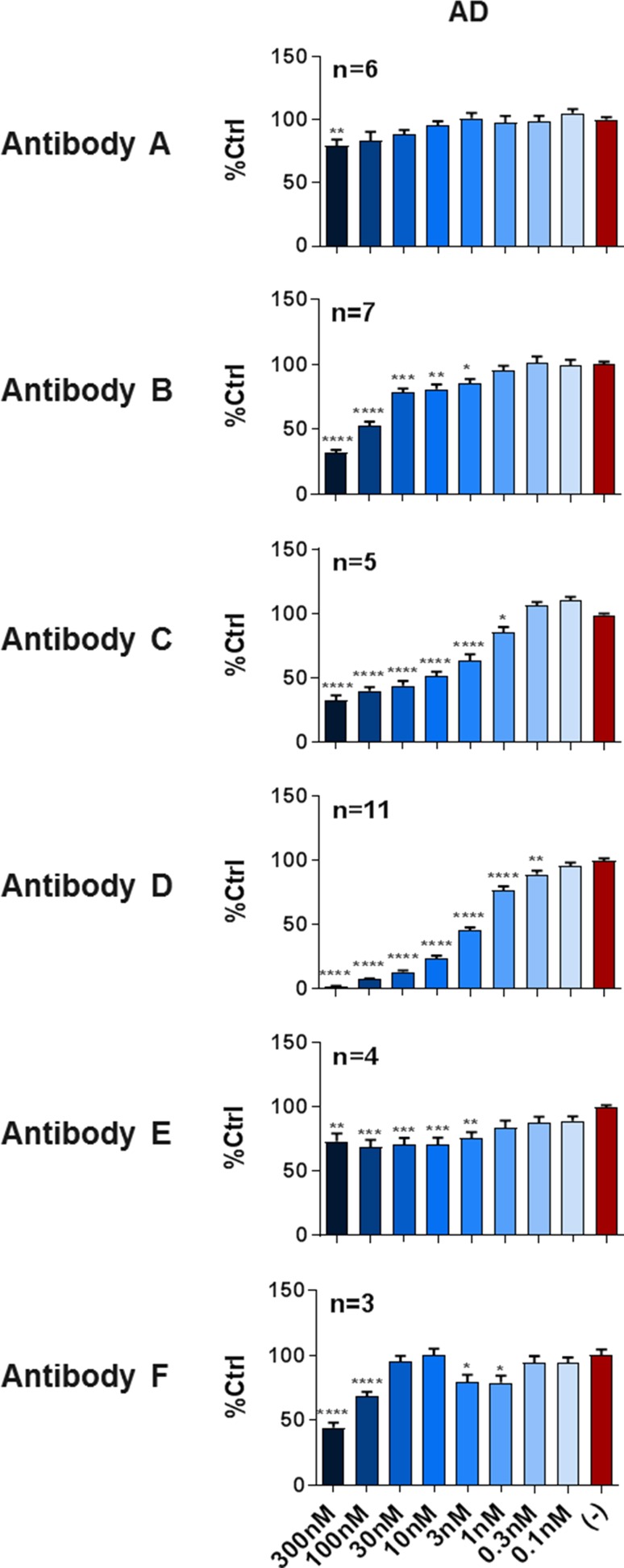
Seeded aggregation can be efficiently blocked by select anti-Tau antibodies. Cells were incubated with human AD seeds in the absence (−) or presence of antibodies at the indicated concentrations and the percentage of seeding relative to vehicle control is plotted (mean ± SEM, at least 3 independent experiments per condition for AD Tau). Generally, Tau antibodies binding the N- (antibodies A and B) or C-termini (antibody E) do not efficiently neutralize Tau seeds from Alzheimer’s disease. While antibody A was essentially inactive in this assay, antibody B did show activity against AD Tau, but only at high concentrations. Phospho-specific antibody C partially neutralized Tau seeds, while antibody D fully and efficiently neutralized Tau seeds. Antibody F showed only partial activity on AD Tau. Data were analyzed by one way ANOVA followed by Dunnett’s post hoc test. *P < 0.05, **P < 0.01, ***P < 0.001, ****P < 0.0001

When tested against AD-PHF Tau, antibodies A and E failed to substantially neutralize seeding activity in a dose-dependent manner (Fig. 7). In contrast, antibody B demonstrated partial activity against AD-PHF Tau, neutralizing seeding activity by 74% (Fig. 7). It should be noted, however, that this effect was seen abruptly and only at the highest doses tested. Neutralization of AD-PHF by antibody C also reached a plateau and failed to fully prevent seeding (Fig. 7). Antibody F only exhibited activity against AD-PHF Tau at the highest concentrations tested (Fig. 7). The most effective antibody tested against AD-PHF Tau was antibody D which fully neutralized seeding activity with an IC50 of 2.9 nM (Fig. 7).

### Antibody D also shows full neutralization activity against Tau seeds from PSP

**Fig. 8:**
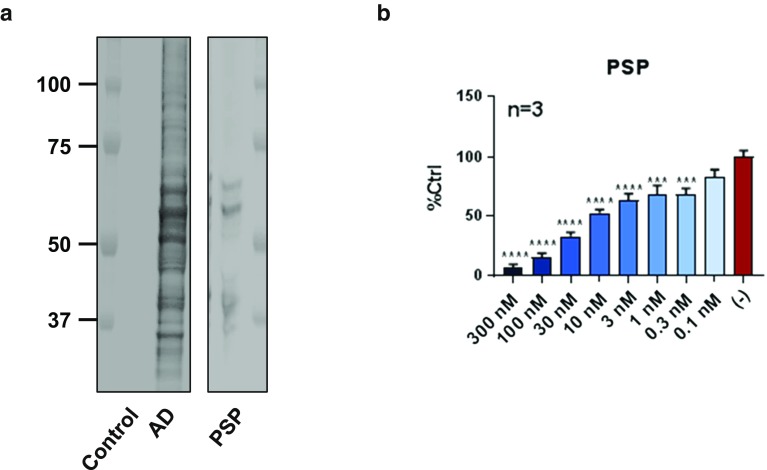
Antibody D efficiently neutralizes Tau seeds from PSP as well as AD. **a** Sarkosyl-insoluble tau from AD and PSP was analyzed by western blot using AT8 to confirm the presence of assembled Tau species and verify inclusion of samples containing the deposition of Tau isoforms appropriate to each disease. Tau from AD migrates as three distinct bands which contain all 6 Tau isoforms, while PSP Tau migrates as two bands which contain only 4R Tau. The Control brain did not contain sarkosyl-insoluble, fibrillar Tau and, therefore, did not display a signal in this experiment. **b** Cells were incubated with human PSP Tau seeds in the absence (−) or presence of antibody D at the indicated concentrations and the percentage of seeding relative to vehicle control is plotted (mean ± SEM, 3 independent experiments per condition). Antibody D fully and efficiently neutralizes Tau seeds from PSP in addition to AD. Data were analyzed by one-way ANOVA followed by Dunnett’s post hoc test. ***P < 0.001, ****P < 0.0001

Tauopathies are characterized by the deposition of assembled Tau species containing different isoforms which are largely specific to individual diseases. Tau deposits in Pick’s disease are formed predominately of three repeat Tau, while those present in PSP are characterized by deposits of four repeat Tau. The deposits in AD are composed of equimolar quantities of three and four repeat Tau. We hypothesized that the inclusion of different Tau isoforms into fibrils could result in conformational variants whereby distinct epitopes might be surface-exposed in only some diseases. To biochemically confirm brain samples used in these experiments contained pathological Tau with deposition of the anticipated isoforms, sarkosyl-insoluble Tau preparations from control brain, PSP, and AD were analyzed by AT8 western blot (Fig. 8a). As expected, three hyperphosphorylated Tau bands migrating between approximately 50 and 64 kDa were observed in the AD preparation, while only two bands corresponding to 4R Tau were observed in the PSP preparation. The migration pattern of the PSP bands corresponded to the pattern previously observed [14]. Once we confirmed that our PSP pool contained fibrillar Tau displaying the expected biochemical profile, we performed a dose titration with antibody D to assess its activity against seeds from this distinct Tauopathy. Antibody D was both fully efficacious and highly potent against PSP Tau seeds in our assay, yielding an IC_50_ of 5.6 nM (Fig. 8b and Table 2).

**Table 2:** Antibody D IC50s and maximum efficacy were similar when either AD or PSP seeds were used to induce assembly of intracellular Tau

	IC_50_ (nM)	SEM	% Inhibition at 300 nM	*N*
AD	2.9	1.1	98	11
PSP	5.6	1.2	94	3

## Discussion

Tau antibodies are an active area of AD drug discovery [25]. They have been developed to address the “Tau spread hypothesis” whereby, an—as yet—uncharacterized extracellular Tau species is released from one cell and taken up by adjacent cells where it is able to seed further pathology through templating [18]. Theoretically such antibodies could be beneficial through a number of mechanisms, however, in the most parsimonious (and most widely accepted) scenario therapeutic antibodies should engage extracellular pathological Tau species in the interstitial fluid (ISF) that underlie the spread of pathology, but are not necessarily required to directly bind intraneuronal Tau. The antibodies would then block the seeding activity of extracellular Tau, most likely by blocking the initial uptake of these seeds [30] and thereby slow down the spread of Tau pathology. Despite the fact that several molecules are currently in the early stages of clinical development, there is no field consensus as to the desired characteristics of such therapeutic antibodies.

We decided to avoid a priori decisions on epitopes and other antibody features, and instead developed a robust and quantifiable cell based seeding and aggregation assay and used this to select the antibodies which were most effective at neutralizing pathology induced by human pathological seeds. In the ideal assay system aged human neurons would be incubated with ISF from tauopathy patients and one would assess Tau seeding activity and its suppression by Tau antibodies at physiological concentrations. Developing such an assay is not practicable, however, beginning with the work of Diamond [15] cell line based assay systems have been established that can be used to detect the seeding activity of various Tau preparations. We set out to design a recombinant cell model, which would be sensitive enough to allow us to seed with patient derived material but also provide us with a robust and quantifiable assay which we could use to select the most efficacious antibody among dozens of potential candidates. To do so, we expressed a full length, untagged Tau construct to avoid any potential artifacts associated with either the expression of short Tau fragments or the use of large fluorescent proteins. Fibrillar Tau does not enter neurons by interacting with a cell specific receptor, but through interactions with cell surface heparin sulfate proteoglycans [19] which are also required for uptake into HEK cells [32, 37]. We therefore believe that HEK293 cells represent a reasonable model system in which to assess seeding and its inhibition by antibodies.

Native human Tau is a complex protein, comprised of six isoforms, furthermore, in diseased conditions the Tau protein exhibits a plethora of post-translational modifications. As this complexity is not readily reproduced using recombinant protein, we opted to test antibodies against AD-PHF. There is currently no complete characterization of the seeding Tau species in human diseases, however, it has been suggested that different Tau “strains” (consisting of different Tau aggregated conformers) may exist and that these may underlie the differences between different tauopathies and that there may even be some heterogeneity within individual tauopathies [29, 35]. We therefore chose to seed with human AD brain material prepared from pooled samples of > 20 individual donors, thus avoiding the potential confound of selecting for activity against only a few individuals with potentially unique “strain” characteristics.

In order to neutralize seeding activity, an antibody must be capable of recognizing epitopes that are accessible on the surface of biologically relevant aggregated Tau species. To ensure that we could monitor antibody neutralization of seeding activity in a format similar to that encountered in vivo, we combined antibodies with Tau seeds in suspension, and added these mixtures directly to cells. We consciously avoided immunoprecipitation of the seeding material with the therapeutic antibody before incubation with cells. Although immunoprecipitation may be helpful in the identification of antibodies that are capable of recognizing native conformations of assembled Tau species, it does not address functional properties required for aggregate neutralization. Using immunoprecipitation as a screening tool also opens the possibility of selecting antibodies that lack sufficient affinity or those unable to achieve the stoichiometry required for activity.

To identify the most effective antibody, we performed a large-scale immunization campaign utilizing a variety of Tau antigens. Although we also performed immunizations using AD-PHF Tau and peptides targeting known disease-associated phospho-epitopes, we were surprised to discover that the most efficacious antibody (antibody D) arose from immunization with recombinant, non-phosphorylated Tau. In fact, the mid-region epitope targeted by antibody D binds equally well to both monomeric and AD-PHF Tau.

At first glance, phosphorylation-specific antibodies would appear to be the most attractive therapeutic candidates. They are widely used to identify Tau pathology by immunohistochemical and biochemical means, and, with the right epitope, they could be designed to specifically target pathological Tau. However, the phospho-specific antibodies tested in our study did not fully neutralize seeding activity of AD-PHF Tau. Indeed, antibody E was entirely inactive; however, this may be attributed to the fact that this antibody has a lower binding affinity than the other antibodies tested in this study. Antibody C, however, is a high-affinity antibody which exhibits good potency in our assay, but interestingly, the titration curve reaches a plateau at less than 60% inhibition. This partial neutralization activity suggests heterogeneity in phosphorylation among individual Tau seeds, with only a proportion of the aggregated Tau being phosphorylated at this epitope. This may be consistent with a step-wise model in which Tau conformational change precedes hyperphosphorylation [40].

Although intraneuronal accumulations of fibrillar Tau are extensively phosphorylated, owing to limits in sensitivity, it is unclear whether extracellular Tau seeds are as well. Indeed, characterization of extracellular Tau [4, 27] has, thus far focused on monomeric species; so, the processing and post-translational modification of aggregated, seed-competent species remain largely undescribed. Extracellular Tau found in human cerebral spinal fluid (CSF) is present almost entirely as an amino-terminal portion of the protein cleaved between residues 222 and 266 [36], irrespective of disease [1, 27]. As this material lacks the microtubule-binding domain (MTBR), it cannot be seed competent but it would act as a decoy for N terminal antibodies, whose effective concentrations would be reduced by binding to an abundant but non-seeding Tau species. Several of the anti-Tau antibodies currently in clinical development bind to a localized region spanning approximately 20 amino acids at the N-terminus of Tau and could, therefore, be impacted by the presence of physiological CSF Tau.

In addition to concerns about non-specific binding to physiological extracellular Tau, we were also surprised to discover that the two reference antibodies A and B were either inactive (A) or showed activity only at high concentrations (B) in our assays. Importantly, in our hands both of these antibodies show high affinity to monomeric Tau and PHF; so, their lack of activity in the functional assay is not a consequence of expression or purification problems. We hypothesize that the amino-terminal region of seed-competent Tau may have limited accessibility in solution either due to conformational alterations or the presence of N terminal truncations [36]. Indeed, we observed substantial quantities of N-terminally truncated AD-PHF Tau by Western blot using antibodies mapping to the central region or carboxy-terminus of Tau. This observation, however, did not fully explain the low-level activity exhibited by antibodies A and B as large proportions of full length Tau were also present. We hypothesize that the region between amino acids 15–30, recognized by antibodies A and B, may not be fully surface exposed on conformationally altered Tau species in solution. Although this contrasts with clear immunogold labeling of N-terminal epitopes [13], structural alterations may occur upon solid surface binding which are not present in solution. Further, linear epitopes have been previously observed to become inaccessible to high-affinity antibodies following conformational conversion of the monomeric, cellular prion protein to infectious, aggregated prions [23, 34].

Antibody A was developed to bind an N-terminal fragment of Tau hypothesized to potentiate beta amyloid accumulation [3], and this is the first description of its activity in a seeded aggregation model. At high doses with antibody B, we did observe limited activity; so, it is, therefore, possible that an optimal seed: antibody stoichiometry may be required for neutralization activity with this antibody. Antibody B was also previously tested against AD-PHF [20]; however, in this instance pre-clearing of the seeding extract with immunoprecipitation was performed. Although several rounds of immunoprecipitation were performed, full neutralization of AD-PHF seeding activity was not achieved. This observation is consistent with our data, and supports a model in which the N-terminus of Tau is not fully available for antibody binding. We also confirm here that antibody B binds both monomeric and PHF Tau with high affinity; however, the values calculated in our SPR experiments are higher than those previously reported. Methodological differences including chip coupling and flow rate are the likely explanations for this apparent discrepancy.

Finally, we tested antibody F, which was derived from the widely used conformation-selective antibody, MC-1. This antibody is capable of binding the N-terminus of monomeric Tau; however, its binding affinity is reported to be increased upon an early conformational change in which the N-terminus makes contact with the centrally located microtubule-binding domain [22]. Although preferential binding for PHF Tau over monomeric Tau was observed by SPR, this antibody only demonstrated partial activity against AD-PHF Tau in our cell assay. Like antibodies A and B, antibody F also requires an intact, accessible N-terminus; so, its activity would, therefore, be affected by proteolytic events removing some or all of these epitopes on seeding species.

Our results clearly demonstrate that high-affinity Tau antibodies show dramatic differences in their ability to block Tau seeding in our in vitro system, suggesting that only some Tau epitopes are accessible on the majority of Tau seeds. The structure of Tau seeds is currently not well understood, but it appears that they are fibrillar species of high molecular weight both in human AD [38] and in transgenic mouse brains [21] and our data are consistent with the idea that there may be substantial heterogeneity in the degree of accessible epitopes.

Like prions, Tau aggregates have been shown experimentally to adopt multiple conformational variants, or strains, which possess distinct biochemical and biological features [29, 35]. How the interplay between post-translational modification and Tau isoform inclusion into fibrils influences higher-order conformation remains incompletely understood and it is as yet unclear which of these features drive the biological diversity of tauopathies. It is accepted, however, that different tauopathies are neuropathologically characterized by the presence of 3R, 4R, or 3R + 4R Tau intracellular inclusions. It is therefore, possible that assembled Tau species from different diseases could present different surface-exposed epitopes. In this scenario, an antibody with promise in AD may not necessarily be active in other tauopathies. We tested this hypothesis by developing a comparable cell assay using seeds from PSP patients. Like AD, PSP seeds were fully neutralized with antibody D suggesting potential efficacy in at least two distinct human diseases.

Recent high-resolution structural data of isolated Tau filaments from Alzheimer’s disease [13] and Pick’s disease [12] have indeed demonstrated clear differences in the residues contained within the core of these types of fibrillar Tau. While the core of Alzheimer’s disease Tau filaments is comprised of residues 306–378 (2N4R Tau numbering), the fibril core of filaments from Pick’s disease contains residues 254–378. In agreement with previously published reports [12, 41], we were unable to seed our 4R Tau cell assay using seeds from Pick’s disease (data not shown) and, therefore, did not test the ability of antibody D to neutralize seeds from this disease. We did, however, confirm binding of filamentous Pick’s disease Tau with antibody D in an ELISA assay (Suppl. Figure 4, Online Resource 1). When loading was normalized to AT8 Tau levels, a decrease in signal was observed with Pick’s disease fibrils compared to Alzheimer’s disease and PSP fibrils. Although antibody D binds outside the Pick fold, decreased ELISA signal suggests that the epitope may still be partially occluded, possibly owing to tight packing and limited flexibility of individual Tau molecules in this region of the filament.

The first structural studies on disease relevant forms of Tau are now starting to appear in the literature, and as more data become available, it may in the future be possible to rationally design therapeutic antibodies. However, as the exact nature of Tau seeds is currently not well understood, we took the pragmatic approach of testing the functional activity of our antibodies against seeds isolated from patient-derived material. Our antibody, in contrast to several of the antibodies currently in clinical development, fully neutralized seeds isolated from both AD and PSP patients. We, therefore, suggest that this antibody offers the opportunity to therapeutically test the Tau spread hypothesis in both of these tauopathies.

## Electronic supplementary material

Below is the link to the electronic supplementary material.

Supplementary material 1 (PDF 422 kb)
