# Supplementary material for: Epitope determines efficacy of therapeutic anti-Tau antibodies in a functional assay with human Alzheimer Tau
Source: Acta Neuropathol. 2018 Sep 20;136(5):729–45. doi: 10.1007/s00401-018-1911-2 (PMC6208734; doi:10.1007/s00401-018-1911-2)
Supplement: Supplementary file 1 — Supplementary material 1 (PDF 422 kb) [file 401_2018_1911_MOESM1_ESM.pdf]

Epitope determines efficacy of therapeutic anti-Tau antibodies in a functional assay with human Alzheimer Tau

Journal: Acta Neuropathologica

Authors: Jean-Philippe Courade[†a], Rachel Angers[†a], Georges Mairet-Coello[a], Nathalie Pacico[a], Kerry Tyson[b], Daniel Lightwood[b], Rebecca Munro[b], David McMillan[b], Robert Griffin[b], Terry Baker[b], Dale Starkie[b], Ruodan Nan[b], Marta Westwood[b], Marie-Laetitia Mushikiwabo[a], Sophie Jung[a], Geofrey Odede[b], Berni Sweeney[b], Andrew Popplewell[b], Gillian Burgess[bc], Patrick Downey[a]* and Martin Citron[a]*

Affiliations:
[a]UCB Biopharma, Chemin du Foriest, 1420 Braine l'Alleud, Belgium.

[b]UCB Celltech, 208 Bath Rd, Slough SL1 3WE, United Kingdom.

[c]Current affiliation: Vertex Pharmaceuticals 86-88 Jubilee Avenue OX14 4RW Milton Park, Abingdon, Oxfordshire United Kingdom.

Corresponding Author:

*To whom correspondence should be addressed:

Patrick Downey patrick.downey@ucb.com UCB Biopharma, Chemin du Foriest, 1420 Braine l'Alleud, Belgium +32-2-386 6497

or Martin Citron martin.citron@ucb.com UCB Biopharma, Chemin du Foriest, 1420 Braine l'Alleud, Belgium +32-2-386 2379

[†]Equal contributors

Supplementary Methods

Peptide blocking

Antibody D was titrated to determine the lowest concentration which elicited a robust signal by western blot. The antibody was then combined with 100-fold molar excess of a blocking peptide and incubated for 2 hours to probe an AD PHF western blot. The peptide used in this experiment (Ac-RTPPKSPSSAKSRLQ-NH_2) was synthesized by Peptide Synthetics (Funtley, United Kingdom).

Assessment of seeding activity in the presence or absence of Lipofectamine

To assess seeding efficiency in the presence of Lipofectamine, HEK293F cells expressing 2N4R P301S Tau were plated at a density of 30,000 cells per well on 96-well plates coated with poly-D-lysine and incubated overnight at 37°C in the presence of 5% CO_2. The following day, AD PHF Tau was titrated in OptiMEM, followed by the addition of 0.5 µl Lipofectamine 2000 in a total volume of 50 µl per well. Cells were incubated for 3 hours at 37°C in 5% CO_2 after which time seeding mixtures were removed and complete medium added for an additional 48 hours of growth. To compare seeding efficiency in the absence of Lipofectamine, AD PHF Tau was titrated in complete medium and added to cells for 48 hours using the conditions outlined above. In each case, after two days of fibrillar Tau treatment, cells were washed in PBS. Lysates were prepared using 45 µl of FRET lysis buffer (Cisbio, France) per well and analyzed according to manufacturer recommendations.

Antibody D sandwich ELISA

White ELISA plates were coated overnight with 0.1µg/ml humanized antibody D in bicarbonate/carbonate coating buffer (Thermo Fisher Scientific). Plates were then blocked with 5% bovine serum albumin (Thermo Fisher Scientific) in PBS-T. Sarkosyl-insoluble Tau was normalized for phospho-Tau using an AT8 TR-FRET kit (Cisbio) prior to ELISA analysis. Assembled Tau species were detected with 0.1µg/ml murinized Antibody D in combination with an anti-mouse secondary antibody conjugated to horse radish peroxidase (Thermo Fisher Scientific). SuperSignal Elisa Femto was used to develop the reaction and chemiluminescent signal was collected using a SpectraMax Paradigm (Molecular Devices).

Supplementary figures

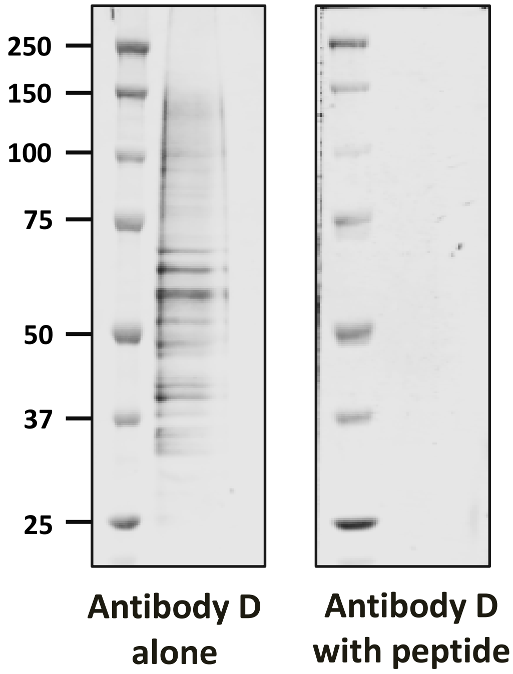

Suppl. Fig. 1. Immunoreactivity of Antibody D was specifically inhibited upon incubation with a blocking peptide. Antibody D was pre-incubated for 1 hour at room temperature in the presence of a 100-fold molar excess of blocking peptide. Antibody binding to full length AD PHF Tau, as well as high and low molecular weight species was specifically prevented by pre-adsorption to the peptide

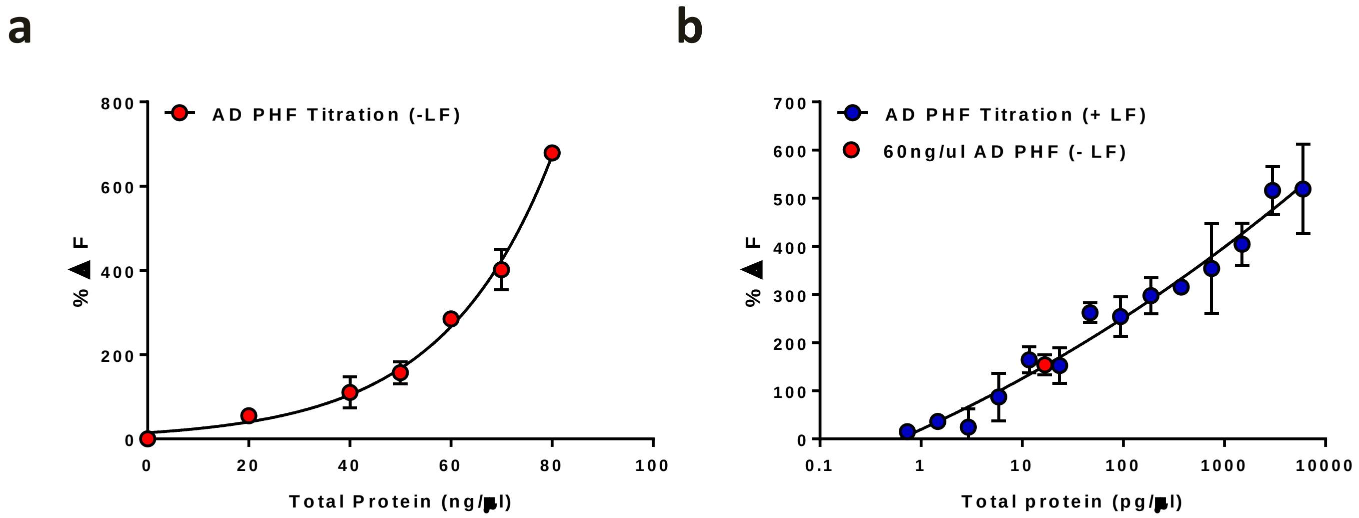

Suppl. Fig. 2. Comparison of seeding efficiency in the presence and absence of Lipofectamine. HEK293 cells expressing P301S tau were treated with increasing concentrations of AD PHF Tau in the **(a)** absence or **(b)** presence of Lipofectamine. Notable differences in efficiency were observed between the methods, and when directly compared in the same experiment **(b)**, an increased efficiency of approximately 2500-fold was observed in the presence of Lipofectamine. Concentrations indicate total protein content as determined by BCA assay. Please note the difference in X axis scale: **(a)** being in ng/µl and **(b)** being in pg/µl

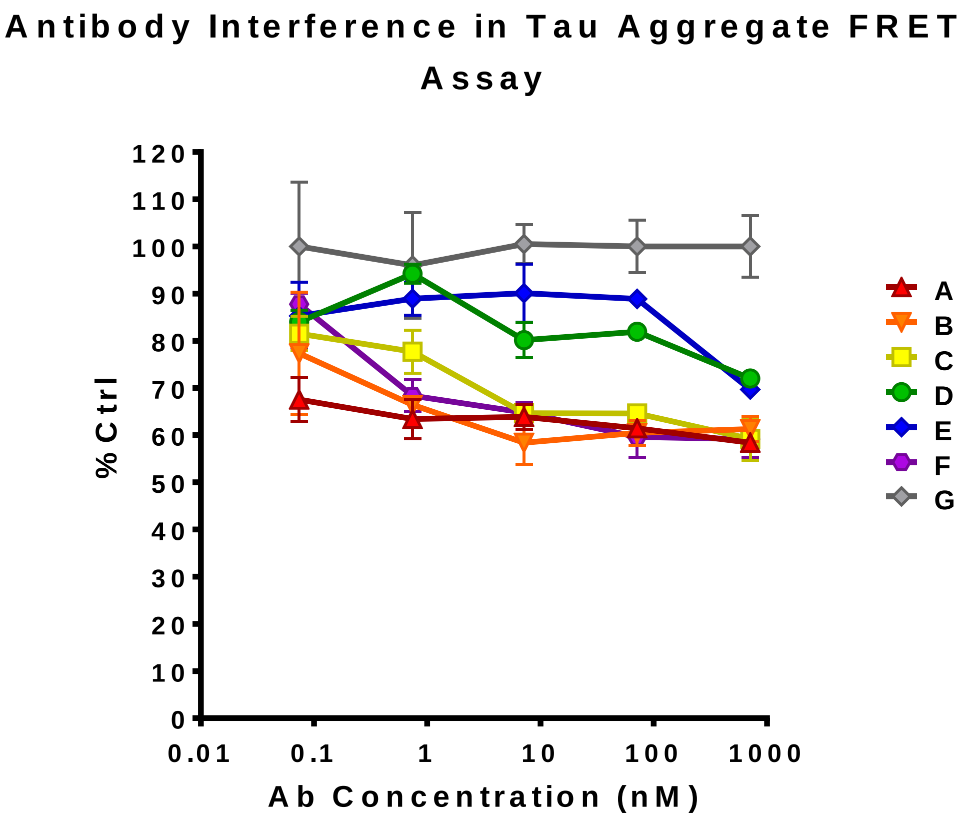

Suppl. Fig. 3. Therapeutic antibody candidates do not specifically interfere with the FRET antibody in the aggregation assay. Tau antibodies were titrated at increasing doses into cell lysate from HEK293 P301S cells seeded with P301S aggregates from end stage transgenic mice. Samples were then analyzed by FRET to test whether Tau antibodies specifically interfered with the antibodies used in the FRET assay to detect aggregation. Although all Tau antibodies exhibited lower signal than control antibody G, none displayed a consistent dose-response indicative of specific assay interference

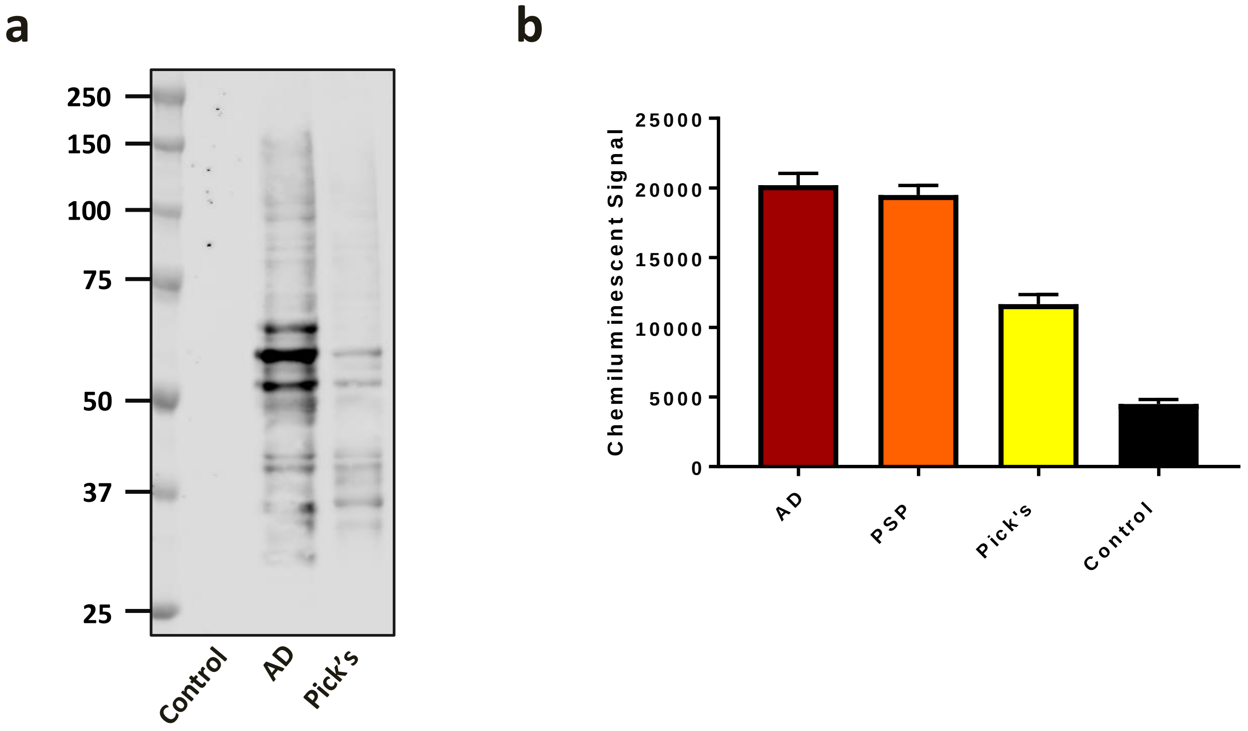

Suppl. Fig. 4. Antibody D binds fibrillar Tau from Pick's disease. (a) Sarkosyl-insoluble Tau from AD, Pick's disease or control brain was assessed using AT8. As anticipated, AD tau was characterized by three predominant bands, while the Pick's disease extract contained only two bands corresponding to 3R Tau. **(b)** A humanized version of antibody D was used to capture sarkosyl-insoluble Tau from AD, PSP, Pick's disease or control brain, then assembled species were detected with a murinized version of the same antibody. Prior to ELISA analysis, Tau samples were normalized to AT8 levels using a commercially available TR-FRET kit (Cisbio, France). ELISA signal was similar for Tau extracted from AD and PSP samples, while signal was decreased for Pick's disease Tau suggesting only partial epitope availability. Three independent experiments were performed and the data shown is a compilation of these; error bars indicate standard error of the mean.
